# Carbon fluxes in ecosystems of Yellowstone National Park predicted from remote sensing data and simulation modeling

**DOI:** 10.1186/1750-0680-6-3

**Published:** 2011-08-11

**Authors:** Christopher Potter, Steven Klooster, Robert Crabtree, Shengli Huang, Peggy Gross, Vanessa Genovese

**Affiliations:** 1 NASA Ames Research Center Mail Stop 242-4, Moffett Field, CA 94035 USA; 2 California State University, Monterey Bay, Seaside, CA 93955, USA; 3 Yellowstone Ecological Research Center, 2048 Analysis Drive, Bozeman, MT 59718, USA

## Abstract

**Background:**

A simulation model based on remote sensing data for spatial vegetation properties has been used to estimate ecosystem carbon fluxes across Yellowstone National Park (YNP). The CASA (Carnegie Ames Stanford Approach) model was applied at a regional scale to estimate seasonal and annual carbon fluxes as net primary production (NPP) and soil respiration components. Predicted net ecosystem production (NEP) flux of CO_2 _is estimated from the model for carbon sinks and sources over multi-year periods that varied in climate and (wildfire) disturbance histories. Monthly Enhanced Vegetation Index (EVI) image coverages from the NASA Moderate Resolution Imaging Spectroradiometer (MODIS) instrument (from 2000 to 2006) were direct inputs to the model. New map products have been added to CASA from airborne remote sensing of coarse woody debris (CWD) in areas burned by wildfires over the past two decades.

**Results:**

Model results indicated that relatively cooler and wetter summer growing seasons were the most favorable for annual plant production and net ecosystem carbon gains in representative landscapes of YNP. When summed across vegetation class areas, the predominance of evergreen forest and shrubland (sagebrush) cover was evident, with these two classes together accounting for 88% of the total annual NPP flux of 2.5 Tg C yr^-1 ^(1 Tg = 10^12 ^g) for the entire Yellowstone study area from 2000-2006. Most vegetation classes were estimated as net ecosystem sinks of atmospheric CO_2 _on annual basis, making the entire study area a moderate net sink of about +0.13 Tg C yr^-1^. This average sink value for forested lands nonetheless masks the contribution of areas burned during the 1988 wildfires, which were estimated as net sources of CO_2 _to the atmosphere, totaling to a NEP flux of -0.04 Tg C yr^-1 ^for the entire burned area. Several areas burned in the 1988 wildfires were estimated to be among the lowest in overall yearly NPP, namely the Hellroaring Fire, Mink Fire, and Falls Fire areas.

**Conclusions:**

Rates of recovery for burned forest areas to pre-1988 biomass levels were estimated from a unique combination of remote sensing and CASA model predictions. Ecosystem production and carbon fluxes in the Greater Yellowstone Ecosystem (GYE) result from complex interactions between climate, forest age structure, and disturbance-recovery patterns of the landscape.

## Background

The Greater Yellowstone Ecosystem (GYE) provides a unique opportunity to study carbon cycles in the western evergreen forests and rangelands of North America. The GYE is the largest remaining continuous wildland area in the United States outside of Alaska. Yellowstone National Park (YNP) is considered to be one of the world's largest intact sub-alpine ecosystem in the northern temperate zone [1]. The absence of historical forest management for timber production in YNP and the extensive wildfires of 1988 combine to make the central GYE a landscape with complex controls on plant production and woody biomass pools (both standing and downed).

Fire has played a major role in influencing the ecological processes and landscape patterns of YNP [2-5]. As reviewed by Kashian et al. [6], the 1988 Yellowstone fires resulted in a 250,000-ha mixed landscape cover mainly of lodgepole pine (*Pinus contorta *var. *latifolia *Englem. ex Wats.), with regrowing sapling densities ranging from fewer than 50 stems ha^-1 ^to more than 500,000 stems ha^-1 ^[7,8]. These fires represented a natural disturbance event that occurs at intervals of 100-300 years in this region [4]. Smaller fires (usually less than 5000 ha) occur more frequently on the Yellowstone landscape during the interval between these large fires [5]. As a result of this fire regime, Yellowstone currently contains a mosaic of young forest stands created by the 1988 fires and small fires that have occurred since 1988, as well as more mature stands of up to 450 years old.

Non-forest cover including mixed grassland, sagebrush, and wetland sedge vegetation in YNP provides critical grazing lands for a unique community of large ungulate herbivores [1]. Two-thirds of the historical winter range for these large ungulate herds is within YNP's northern and central rangelands [9,10]. Natural fire return intervals in Yellowstone may be as short as 20-25 years for shrub and grasslands in the Northern Range [11].

Previous studies of plant production patterns in YNP have pointed to soil types and elevation gradients as important determinants [12]. Turner et al. [7] reported that most of the variation in plot-based aboveground productivity and leaf area index (LAI) in post-fire lodgepole pine stands measured across YNP was explained by sapling density, with minor variation explained by the abiotic factors, elevation and soil class. Analysis of environmental variables on CASA model NPP (at 8-km spatial resolution across YNP) by Crabtree et al. [13] revealed that soil properties had the strongest influence on NPP spatial patterns, followed by solar radiation. The diverse topographic variation in YNP with large amounts of shade intolerant lodgepole pine favors NPP responding to solar radiation. The next strongest influence on NPP reported by Crabtree et al. [13] was precipitation. However, precipitation and temperature were spatially correlated across YNP and collectively represent the influence of climate in the model. The hypothesis was offered by Crabtree et al. [13] that gradients in temperature, precipitation, and wildfire severity have had a lesser effect on NPP in YNP during the last decade than either soil fertility or solar radiation patterns.

Previous field measurements of carbon cycling in forest ecosystems of YNP have revealed that stand structure and woody biomass increment are closely linked [6,14,15]. Aboveground NPP and belowground carbon allocation increase with forest age to around 250 years of pine stand growth [16,17].

The objective of this study was to quantify the carbon cycle of all ecosystems in YNP over the period 2000-2006, including the residual effects of the wildfires of 1988 on forest biomass decomposition and regeneration rates. We applied the CASA model [18,19] using MODIS EVI inputs at 250-meter spatial resolution. Our modeling framework has been designed to estimate historical as well as current monthly patterns in plant carbon fixation, living biomass increments, nutrient allocation, litter fall and decomposition, long-term decay of downed woody pools, soil CO**_2 _**respiration, and soil nutrient mineralization before, during, and after disturbance events such as wildfires. To our knowledge, this is the first study to take full advantage of 250-meter MODIS land products together with airborne remote sensing to make annual net biome production (NBP) estimates for YNP.

The three main study questions that could be uniquely addressed with MODIS satellite observations (years 2000 - 2006) and the CASA ecosystem model were:

• What is the year-to-year variability in net primary production as represented in different landscape areas (one to several km^2 ^in size) of YNP?

• What is the carbon balance across all ecosystems of YNP 20 years after the wildfires of 1988?

• What are the rates of vegetation production in the 1988 burned areas of YNP?

## Methods

### Study area

**Figure 1:**
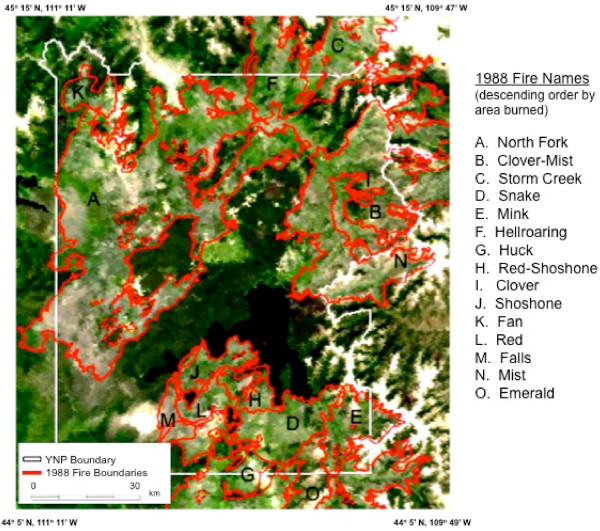
**Yellowstone study area boundaries, shown with a MODIS true-color composite image and 1988 fire area labels**.

The study area was YNP, located in Wyoming (96%), Montana (3%), and Idaho (1%) and its Northern Range [11] (Figure 1; NW corner coordinates: 45°15' N, 111°12' W; SE corner coordinates: 44°5' N, 109°49' W). The Park area has elevations ranging from 1540 m to 3760 m. Nearby mountain ranges include the Gallatin Range to the northwest, the Beartooth Mountains in the north, the Absaroka Range to the east, and the Teton Range and the Madison Range to the southwest and west. The Continental Divide of North America runs diagonally through the southwestern part of the Park. The divide is a topographic feature that separates Pacific Ocean and Atlantic Ocean water drainages. About one third of the park lies on the west side of the divide.

The climate is generally cool and dry with mean January and July temperatures of -11.4°C and 10.8°C, respectively, and mean annual precipitation of 56.3 cm [20]. Winters are long and cold, lasting from mid-November to mid-March. Summers are short and often dry, usually lasting from July through August. Average annual snow depth is around 33 cm [21].

Soils in YNP at higher elevations are largely nutrient-poor rhyolites and andesites with low water-holding capacity [22]. Valley bottoms and floodplains contain glacial out-wash and alluvial soils that are higher in nutrients and water-holding capacity. Soils derived from rhyolitic parent materials typically are coarser and have fewer base cations and lower water-holding capacity than soils derived from andesite or lacustrine sediments. Lacustrine sediments typically have the highest silt and clay content, base cations, and water-holding capacity [5].

Most of the forests of YNP consist of five conifer tree species [23]: lodgepole pine, whitebark pine (*Pinus albicaulis*), Douglas fir *(Pseudotsuga menziesii*), Engelmann spruce (*Picea engel-mannii*), and subalpine fir (*Abies lasiocarpa*). Elevation and soil fertility are considered to be the two most important abiotic gradients controlling forest vegetation on the subalpine plateaus [5,13]. Non-forest vegetation is divided into four major groups: grassland, sagebrush steppe (shrubland), wet sedge and willow meadow, and alpine meadow. The distributions of these vegetation types are influenced strongly by elevation. Big sagebrush grows in dry to moderately moist areas at middle and lower altitudes, such as in the Lamar River Valley. Silver sage grows in wetter areas higher than 2130 m, for example, in the Hayden and Pelican Valleys. Alpine meadows are present at elevations higher than 3050 m. Sedge marshes and other wetland vegetation thrive in areas of year-round standing water at various elevations throughout YNP [23].

**Figure 2:**
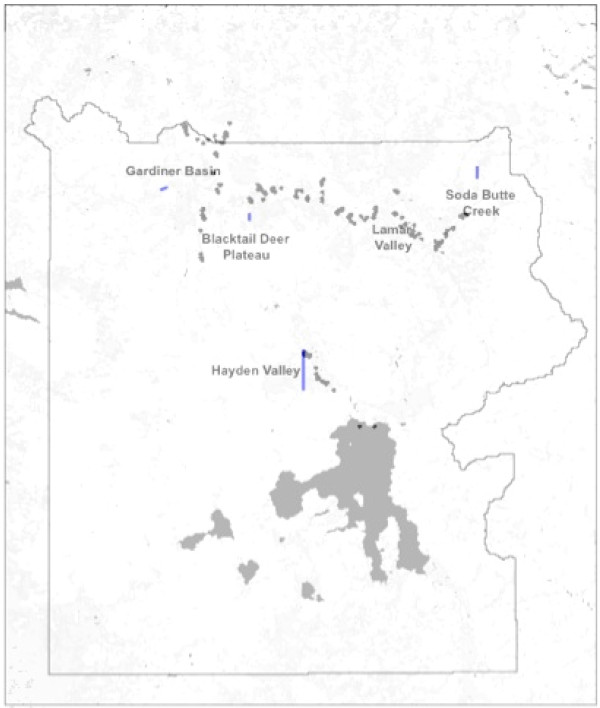
**Landscape transects selected within YNP, together with field survey plot locations (black dots) in grasslands of the Gardiner Basin, Lamar and Hayden Valleys**.

For this study, we selected five landscapes (Figure 2), none of which had been severely affected by wildfires during the 1988 fires, for validation and characterization of CASA predictions of (unburned ecosystem) carbon cycles within YNP. The selected landscape areas are described briefly below in terms of vegetation and topography.

#### Gardiner Basin

The Gardiner River Basin in the northwestern corner of YNP consists of a variety of sagebrush habitats including the Wyoming big sagebrush (*Artemisia tridentata wyomingensis*) and blue bunch wheatgrass (*Agropyron spicatum*) that is found primarily at lower elevations in the basin. Mountain big sagebrush (*A. t. vaseyana*) - Idaho fescue (*Festuca idahoensis*) habitat type dominates the majority of Gardiner Basin [24]. The landscape has relatively mild winter conditions compared to ranges further inside YNP, owing to its lower elevation and windswept slopes, which affords reliable winter foraging for ungulates. The selected transect of four 250-meter series areas examined within the Gardiner Basin landscape ranged moderately in elevation, from 2320 m to 2400 m.

#### Blacktail Deer Plateau

The Blacktail Deer Plateau is an important part of the northern winter range of YNP. The area consists of approximately 100 km^2 ^of moderately sloping terrain (elevation 2000-2300 m). Sagebrush (*Artemisia *spp.) mixed with grasslands and scattered aspen (*Populus tremuloides*) clones occur across much of the plateau. These upland areas are interspersed with willow and sedge (*Carex *spp.), plus grass meadows in swales and riparian zones. The transect of four 250-meter areas examined within this landscape ranged moderately in elevation, from 2256 m to 2291 m.

#### Soda Butte Creek Drainage

Soda Butte Creek is located west of Cache Creek across the forested drainage divide formed partly by Mount Norris ion the northeastern corner of YNP. The Creek flows for a length of approximately 30 kilometers from its headwaters near Cooke City, MT, just outside northeast corner of YNP, until it empties into the Lamar River. Forest cover in the drainage basin is predominantly lodgepole pine giving way to subalpine fir (*Abies lasiocárpa*), Douglas-fir (*Pseudotsuga menziesii*), and Engelmann spruce (*Picea engelmánnii*). Owing to the mountainous terrain of the drainage, the transect of four 250-meter areas examined within this landscape ranged widely in elevation from 2209 m to 2687 m.

#### Lamar River Valley

The Lamar River is a tributary of the Yellowstone River, approximately 48 kilometers long, located entirely within YNP. The Lamar River headwaters are located in the Absaroka Range, on the eastern edge of the Park. The main channel is joined by many tributary streams, including Soda Butte Creek and Slough Creek, and empties into the Yellowstone River near Tower Junction. Pleistocene outwash deposits have created an expansive valley floor that ranges from 1 to 2 km in width. Grassland and sagebrush - Idaho fescue *(Festuca idahoensis*) vegetation types occur on most of the northern winter range of the Lamar Valley. Herbaceous cover is composed also of blue bunch wheatgrass (*Agropyron spicatum*), june grass (*Koeleria macrantha*), needle grasses (*Stipa comata*), basin wild rye *(Elymus cinereus*), blue grasses (*Poa spp*.), and various forbs.

#### Hayden Valley

Hayden and Pelican Valleys makes up a large portion of the central winter range for grazing animal in YNP. The valleys are situated on ancient lakebed left over from the last Ice Age and the soils there are still influenced by deposits of lake sediment. As in the Lamar Valley, the vegetation in this landscape is dominated by a mix of sagebrush and Idaho fescue cover. The transect of four 250-meter areas examined within the Hayden Valley landscape ranged in elevation from 2356 m to 2402 m.

### Background on CASA Carbon Modeling

The launch of NASA's Terra satellite platform in 1999 with the Moderate Resolution Imaging Spectroradiometer (MODIS) instrument on-board initiated a new era in remote sensing of the Earth system with promising implications for carbon cycle research. Direct input of satellite vegetation index "greenness" data from the MODIS sensor into ecosystem simulation models is now used to estimate spatial variability in monthly net primary production (NPP), biomass accumulation, and litter fall inputs to soil carbon pools. Global NPP of vegetation can be predicted using the relationship between leaf reflectance properties and the absorption of photosynthetically active radiation (PAR), assuming that net conversion efficiencies of PAR to plant carbon can be approximated for different ecosystems or are nearly constant across all ecosystems [25,26].

Operational MODIS algorithms generate the Enhanced Vegetation Index (EVI) [27] as global image coverages from 2000-present. EVI represents an optimized vegetation index, whereby the vegetation index isolines in red and near infra-red spectral bands are designed to approximate vegetation biophysical isolines derived from canopy radiative transfer theory and/or measured biophysical-optical relationships [28]. EVI was developed to optimize the greenness signal, or area-averaged canopy photosynthetic capacity, with improved sensitivity in high biomass regions. The EVI has been found useful in estimating absorbed PAR related to chlorophyll contents in vegetated canopies [27] and has been shown to be highly correlated with processes that depend on absorbed light, such as gross primary productivity (GPP) [28,29].

As documented in [30], the monthly NPP flux, defined as net fixation of CO_2 _by vegetation, is computed in NASA-CASA on the basis of light-use efficiency [31]. Monthly production of plant biomass is estimated as a product of time-varying surface solar irradiance, Sr, and EVI from the MODIS satellite, plus a constant light utilization efficiency term (emax) that is modified by time-varying stress scalar terms for temperature (T) and moisture (W) effects (Equation 1).(1)

The e_max _term was set uniformly at 0.55 g C MJ^-1 ^PAR, a value that derives from calibration of predicted annual NPP to previous field estimates [18]. This model calibration has been validated globally by comparing predicted annual NPP to more than 1900 field measurements of NPP [19,32]. Interannual NPP fluxes from the CASA model have been reported [33] and validated against multi-year estimates of NPP from field stations and tree rings [34]. Our NASA-CASA model has been validated against field-based measurements of NEP fluxes and carbon pool sizes at multiple forest sites [35-37] and against atmospheric inverse model estimates of global NEP [18].

The T stress scalar is computed with reference to derivation of optimal temperatures (Topt) for plant production. The Topt setting will vary by latitude and longitude, ranging from near 0°C in the Arctic to the middle thirties in low latitude deserts. The W stress scalar is estimated from monthly water deficits, based on a comparison of moisture supply (precipitation and stored soil water) to potential evapotranspiration (PET) demand using the method of [38].

The 2001 National Land Cover Dataset (NLCD) 30-meter map from the U. S. Geologic Survey was aggregated to 250-meter pixel resolution and used to specify the predominant land cover class for the W term in each pixel as either forest (evergreen or deciduous), shrubland, grassland, or other classes such as open water or urbanized area. The NLCD product is derived from 30-meter resolution Landsat satellite imagery and has been shown to have a high level of accuracy in the western United States [39]. Monthly mean surface air temperature and precipitation grids for model simulations over the years 2000-2006 came from PRISM products [40]. Monthly mean inputs of solar radiation flux to the model were derived from Huang et al. [21]. Soil settings in the model for texture classes (fine, medium and coarse) and depth to bedrock for maximum plant rooting depths were assigned to the YNP map product of Rodman et al. [22].

Carbon accumulation rates in forest biomass at the stand level are a function of both growth and mortality of trees, related to other factors such as soils and microclimate. For the CASA model, Potter and Klooster [41] reported that these processes could be expressed in terms of the mean residence time (τ, in years) of carbon in the aboveground wood tissue pools. Tissue allocation ratios (α percent of NPP) were expressed in a similar manner, based on estimates from the global ecosystem literature. These default forest values for τ (50 years) and α (45%) together determine the model's estimation of potential accumulation rates of forest biomass, both living and dead. These potential accumulation rates of woody biomass are based on the assumption of forest growth to mature stand status, but are subject to validation and readjustment based on comparisons to inventory measurements.

Evapotranspiration in CASA is connected to water content in the soil profile layers (Figure 2), as estimated using the CASA algorithms described by Potter et al. [18]. The soil model design includes three-layer (M1-M3) heat and moisture content computations: surface organic matter, topsoil (0.3 m), and subsoil to rooting depth (1 to 2 m). These layers can differ in soil texture, moisture holding capacity, and carbon-nitrogen dynamics. Water balance in the soil is modeled as the difference between precipitation or volumetric percolation inputs, monthly estimates of PET, and the drainage output for each layer. Inputs from rainfall can recharge the soil layers to field capacity. Excess water percolates through to lower layers and may eventually leave the system as seepage and runoff.

Based on plant production as the primary carbon and nitrogen cycling source, the NASA-CASA model is designed to couple daily and seasonal patterns in soil nutrient mineralization and soil heterotropic respiration (Rh) of CO_2 _from soils. Net ecosystem production (NEP) can be computed as NPP minus Rh fluxes, including the effects of wildfires and other localized disturbances or vegetation regrowth patterns on carbon fluxes. The soil model uses a set of compartmentalized difference equations. First-order decay equations simulate exchanges of decomposing plant residue (metabolic and structural fractions) at the soil surface. The model also simulates surface soil organic matter (SOM) fractions that presumably vary in age and chemical composition. Turnover of active (microbial biomass and labile substrates), slow (chemically protected), and passive (physically protected) fractions of the SOM are represented. Along with moisture availability and litter quality, the predicted soil temperature in the surface (M1) layer controls SOM decomposition.

The soil carbon pools were initialized to represent storage and flux conditions in near steady state (i.e., an annual NEP flux less than 0.5% of annual NPP flux) with respect to mean land surface climate recorded for the period 1999-2000. This initialization protocol was found to be necessary to eliminate any notable discontinuities in predicted NEP fluxes during the transition to our model simulation years of interest prior to MODIS EVI availability. Initializing to near steady state does not, however, address the issue that some landscapes may not in equilibrium with respect to net annual carbon fluxes, especially when they are recovering from past disturbances such as wildfires.

### Validation of CASA Aboveground NPP Estimates

During the summer growing seasons of 2008 and 2009, 128 non-riparian field plots 35 meters in diameter were surveyed in grasslands of the Gardiner Basin, Lamar Valley and Hayden Valley (Figure 2) for total production of aboveground herbaceous biomass. YNP is a protected area with many dangerous wild animals (e.g. wolf, bear, and bison), so it is required that any field teams working there avoid specific wildlife management areas, remain invisible to Park visitors, and use only non-destructive sampling methods. The short growing season further restricts field surveys to be completed within four months. For all these reasons combined, Huang et al. [42] adopted a time-saving and cost-effective ocular estimation method for these aboveground biomass measurements, which is a widely employed method for vegetation evaluation [43] and shown to have high accuracy and precision [44]. Ocular estimates can nevertheless be influenced by observer bias and person-to-person variability [45]. For this reason, at least four persons together conducted the field surveys and the average of their biomass estimates was computed for each plot.

Average aboveground production estimated from grassland field survey plots was 99.7 g C m^-2 ^yr^-1 ^with a CV = 88% (CV is the coefficient of variation; defined as the 100 times the ratio of the standard deviation to the mean). Based on a conversion factor of 0.45 for aboveground biomass carbon as a fraction of total (above and below-ground) NPP from the model for non-riparian herbaceous cover [45]. CASA average aboveground production was estimated at 90.6 g C m^-2 ^yr^-1 ^with a CV = 25% for these same 128 field plot locations. Although there was a high level of variance for the plot survey estimates of aboveground production, CASA predicted the measured mean value within about 10% across a wide variety of grassland ecosystems types in YNP.

The spatial layer for coarse woody debris in burned areas of the GYE from Huang et al. [46] was used for the first time in this paper for computing post-fire carbon decomposition emissions of CO_2_. Our coarse woody debris remote sensing product was validated to have an accuracy level of 85% compared to field plot measurements throughout YNP forested sites. This layer has been validated as well to separate downed versus standing dead wood pools.

We note that none of the field measurement data from previous forest carbon cycling studies in YNP [6,7,14-17,47] could be made available to us (upon request) with precise geographic locations and with matching time periods of measurement to CASA model predictions for 2001-2006. Measurements of aboveground forest production [7,47] are not comparable to total forest (above- and belowground) NPP model predictions. Furthermore, carbon measurement data from pre-2000 field measurements for post-1988 burned forest areas cannot be used for validation of CASA model NPP predictions for 2001-2006, because the rate of sapling regrowth since 1988 has been changing rapidly.

## Results

### Carbon Cycling at Landscape Scales

**Figure 3:**
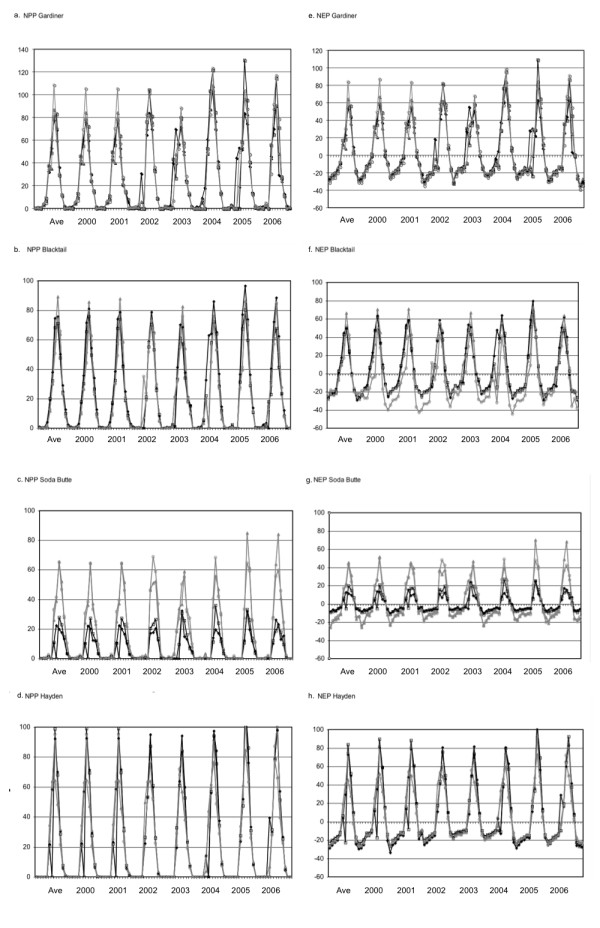
**CASA model monthly NPP (a-d) and NEP (e-h) for the landscape transects selected within YNP for the years 2000 to 2006**. Transects series points were composed of four 250-meter resolution grid cell results falling along a straight line within the landscape area. Points in the Soda Butte Creek series ranged from a high elevation of 2687 m (Series 1) down to 2209 m (Series 4), which was the only transect with a strong elevational gradient.

CASA model predictions of seasonal and inter-annual NPP and NEP were characterized within the selected landscape transects of YNP (Figure 3). Comparisons of annual NPP results showed that 2002 and 2003 were the lowest production years in the Gardiner Basin, on Blacktail Deer Plateau and in Hayden Valley (commonly at NPP less than 250 g C m^-2 ^yr^-1^), whereas 2004 was the lowest production year on Soda Butte Creek drainage at 133 g C m^-2 ^yr^-1 ^(Figure 3a-d). Along all the transects, 2005 and 2006 were generally the most productive years in the time series, with annual NPP varying between 280 g C and 345 g C m^-2 ^yr^-1 ^in the mixed sagebrush-grasslands of the Gardiner Basin, Blacktail Deer Plateau, and Hayden Valley. The more heavily forested Soda Butte Creek transect covered a higher elevation gradient than the other transects and ranged in NPP from 145 to 158 g C m^-2 ^yr^-1 ^in the most productive years of 2005 and 2006.

Variability among predicted annual NPP values within each landscape transect was expressed in terms of yearly sample CVs. Variability in annual NPP in the Gardiner Basin and Hayden Valley was consistently low, with CVs between 4 and 11%. Annual NPP on Blacktail Deer Plateau showed its lowest variance (CV = 6%) in 2002 and its highest variance (CV = 21%) in 2004. Variability in annual NPP across the Soda Butte Creek transect was consistently the highest, with CVs around 50% for every year.

Comparisons of CASA annual NEP results (Figure 3e-h) showed that mixed sagebrush-grassland ecosystems in the Gardiner Basin and in Hayden Valley were consistent yearly sinks for atmospheric CO_2_, at rates of between +50 to +90 g C m^-2 ^yr^-1^. Estimated annual NEP was highest in the Gardiner Basin during 2004 at +130 g C m^-2 ^yr^-1^. Annual NEP was more variable on Blacktail Deer Plateau, with an estimated net source flux of CO_2 _to the atmosphere of -7 g C m^-2 ^yr^-1 ^in 2002 changing to a net sink flux of +31 g C m^-2 ^yr^-1 ^in 2003. Annual NEP for other years on Blacktail Deer Plateau was estimated at net sink flux of around +23 g C m^-2 ^yr^-1^. The higher elevation forested Soda Butte Creek ecosystems were also variable from year to year in terms of NEP flux estimates, ranging from a small net sink of +2 g C m^-2 ^yr^-1 ^in 2004 to larger net sink flux of +31 g C m^-2 ^yr^-1 ^in 2006.

Interannual variation in NPP and NEP can be explained in part by climate input variations to the model. The warmest summer months in the seven-year time series were recorded in 2003, particularly on the Northern Range of the Yellowstone, whereas the coolest summer months were recorded in 2004 and 2005. Annual precipitation was highest in the years 2003 and 2005, where annual precipitation was well below-average in 2001 and 2002. These results imply that the relatively cooler and wetter summer growing seasons were the most favorable for annual plant production and net ecosystem carbon gains in these landscapes of YNP.

### NPP Patterns Across YNP

Annual NPP can vary substantially by vegetation class, which is the result of many abiotic and biotic factors in YNP, including adaptations to (1) harsh winter climates (2) soil fertility and moisture regimes and (3) elevation and topography (Crabtree et al., 2009). These adaptations were reflected in predicted NPP per unit area (m^2^) averaged by vegetation class (Table 1a), which in order from highest to lowest, was: deciduous forest (mainly aspen, *Populus tremuloides*), wooded grassland, mixed forest, shrubland (sagebrush), evergreen forest, Ggrassland, and barren land. When summed across the vegetation class areas, the predominance of evergreen forest and shrubland (sagebrush) cover was evident however, with these two classes together accounting for 88% of the total annual NPP for the Yellowstone study area of 2.5 Tg C yr^-1 ^(1 Tg = 10^12 ^g = 1 million metric tons).

**Table 1:** CASA annual net primary production (NPP) for the Yellowstone study area by (a) vegetation classes, (b) soil texture classes, and (c) 1988 burned areas.

Class Name	Area (m^2^)	Minimum	Maximum	Mean	Standard Deviation
a. NLCD Vegetation					
Water	4.08E+08	0	0	0	0
Barren	5.33E+07	2.4	245.9	55.1	43.6
Grassland	1.89E+09	0.0	467.0	143.3	72.5
**Evergreen Forest**	**6.25E+09**	**1.2**	**431.5**	**182.3**	**50.9**
**Shrubland**	**5.90E+09**	**9.1**	**481.9**	**184.5**	**53.5**
Mixed Forest	6.25E+04	233.8	233.8	233.8	0.0
Cultivation	1.33E+07	70.9	477.6	245.3	69.3
Wooded Grassland	9.03E+07	34.6	389.1	251.7	49.9
Deciduous Forest	1.88E+05	226.1	347.1	271.1	54.1
TOTAL Land	1.42E+10			195.9	
					
b. YNP Soil Texture					
Coarse	2.66E+09	0.0	413.2	191.0	47.2
**Medium-Coarse**	**7.22E+09**	**0.0**	**481.9**	**179.0**	**56.8**
Medium	2.94E+09	0.0	477.6	192.9	54.4
Medium-Fine	3.24E+07	61.9	334.3	205.3	48.9
Fine	1.17E+07	89.7	214.9	141.4	22.9
					
c. FIRE Area 1988					
North Fork	2.14E+09	0.0	400.5	194.5	48.5
Clover-Mist	1.17E+09	0.0	353.2	155.0	50.1
Storm Creek	5.09E+08	0.0	360.0	159.6	59.0
Snake	4.68E+08	0.0	406.2	190.1	53.6
Mink	4.23E+08	0.0	453.2	168.1	60.3
Hellroaring	4.13E+08	36.9	340.2	189.0	46.4
Huck	2.33E+08	48.5	356.8	195.5	41.0
Red-Shoshone	1.39E+08	0.0	387.2	201.1	48.6
Clover	1.28E+08	0.0	271.2	172.0	36.4
Shoshone	1.28E+08	0.0	335.4	211.0	44.1
Fan	1.11E+08	37.3	346.1	188.3	46.6
Red	9.89E+07	0.0	348.0	182.0	53.3
Falls	6.60E+07	0.0	259.7	159.0	32.9
Mist	1.94E+07	51.7	229.1	141.9	30.8
Emerald	5.38E+06	83.4	269.8	187.7	32.4
Stormcreek	2.31E+06	64.1	279.3	177.7	54.8
TOTAL Burned Land	6.05E+09			179.5	

All NPP estimates are in units of g C m^2 ^yr^-1^. Predominant classes by area coverage are in bold print. Fires are sorted by total area burned.

Aboveground production (ANPP) measurements reported by Turner et al. [7] for post-fire lodgepole pine stands in YNP averaged 280 g C m^-2 ^yr^-1 ^(no estimate of variance reported). Lodgepole pine NPP measurements reported by Litton et al. [48] for 110 year-old stands in YNP averaged 240 g C m^-2 ^yr^-1 ^(with a standard error of 20 g C m^-2 ^yr^-1^). Both of these measured estimates are well within the typical range of total forest NPP (above- and belowground) from CASA models predictions for conifer forests of 180 - 430 g C m^-2 ^yr^-1 ^(Table 1).

**Figure 4:**
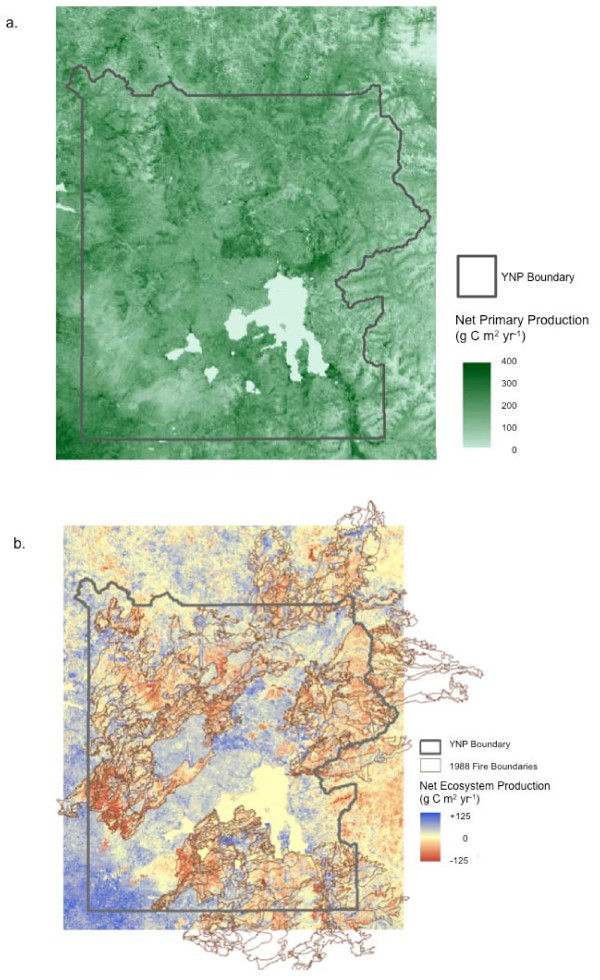
**CASA model annual (a) NPP and (b) NEP (2006) for the Yellowstone study area**. All estimates are in units of g C m^2 ^yr^-1^, with negative NEP values (red) representing net source fluxes of CO_2 _to the atmosphere and positive NEP values (blue) representing net sink fluxes of CO_2 _from the atmosphere to the ecosystem.

Examined by landscape areas, mean annual NPP was highest (around 300 g C m^-2 ^yr^-1^) in the Gardner River Basin, in Hayden and Pelican Valleys, along the Yellowstone River southeast of Yellowstone Lake, and in the Falls River Basin in the extreme southwest corner of YNP (Figure 4a). Mean annual NPP was lowest (less than 100 g C m^-2 ^yr^-1^) in high elevation areas (above 2500 m) of the Madison Plateau (45°23' N, 110°57' W) and the Pitchstone Plateau (44°14' N, 110°47' W). These areas in the southwest corner of YNP are dominated by glaciated rubble and alluvial deposits derived from rhyolite [22].

When computed for CASA soil texture classes, predicted average NPP per unit area (m^2^) was highest for medium-fine textures (205 g C m^-2 ^yr^-1^) and lowest for fine textured soils (141 g C m^-2 ^yr^-1^), with all coarser texture soil classes making up the middle range of annual NPP (Table 1b). Although the medium-fine and fine texture soil classes covered relatively small areas of YNP, they are of interest due to their different locations - the medium-fine textured soils located in the extreme south of YNP along the Snake River (45°15' N, 111°12' W) and the fine texture soils located in the extreme north of YNP along the Gardner Canyon (45°15' N, 111°12' W).

CASA results showed that there were several 1988 burned areas over which mean annual NPP was higher than 250 g C m^-2 ^yr^-1^, implying that vegetation cover in these areas was recovering rapidly from the impacts of the 1988 wildfires. Areas in this rapid recovery category (all greater than 350 hectares) were found within the Mink Fire (44°3' 9" N, 111°15' 37"W), the North Fork Fire (44°41' 13" N, 110°57' 38"W and 44°36' 22" N, 111°4' 36"W), the Clover-Mist Fire (44°34' 54" N, 110°13' 33"W), and the Snake Fire (44°18' 28" N, 110°17' 24"W) boundaries. Burned areas with the highest overall (mean) NPP were the Red-Shoshone and Mist Fires at more than 200 g C m^-2 ^yr^-1^.

Burned areas with the lowest overall (mean) NPP were the Hellroaring, Mink, Stormcreek, and Falls Fires at less than 150 g C m^-2 ^yr^-1 ^(Table 1c). Each of these burned areas with low average productivity was observed to have sub-areas with near zero annual NPP. It is probable that these low production areas are presently covered by a low density of pine saplings that are growing slowing on relatively infertile soils, or are covered predominantly by herbaceous annual grasslands with low pine sapling regeneration.

### Net Ecosystem Production for YNP

#### Refining coarse woody debris inputs

Coarse woody debris (CWD) is a vital component of forest ecosystems, important for carbon and nutrient cycling, tree regeneration following fire, and wildlife habitat. Both the quantity, defined as biomass per unit area tons ha^-1^, and quality, defined as the proportion of standing dead logs to the total CWD quantity, are important for carbon accounting in a disturbed area such as YNP. MODIS data used as inputs to the CASA model cannot detect CWD pools and without direct measurements of CWD across the landscape, and carbon models cannot accurately estimate the effect of stand-replacing disturbances (e.g. the 1988 wildfires) without accounting for the residual biomass pools left in place by loss of the previous forest stand at any given location.

Huang et al. [46] used remote sensing methods to classify and map spatially explicit structural characteristics of CWD in the Yellowstone post-fire ecosystem. A total of 21 AirSAR scenes were acquired in 2003 and an extensive AVIRIS hyperspectral image was acquired on September 25, 2006 for this analysis. As validation of their CWD image classification method, Huang et al. [46] conducted field surveys on 186 plots that were made in summer of 2007 using the line intersect method to inventory downed CWD. CWD volume was calculated according to the methods of Van Wagner [49]. Huang et al. [46] also surveyed the standing CWD and calculated the standing CWD fraction (versus downed CWD) in each plot. The result of this fusion analysis of radar and hyperspectral imagery showed that CWD classified into two categories of ≤ 60 t biomass/ha and ≥ 60 t biomass/ha had an accuracy level of 85% compared to field plot measurements throughout YNP.

**Figure 5:**
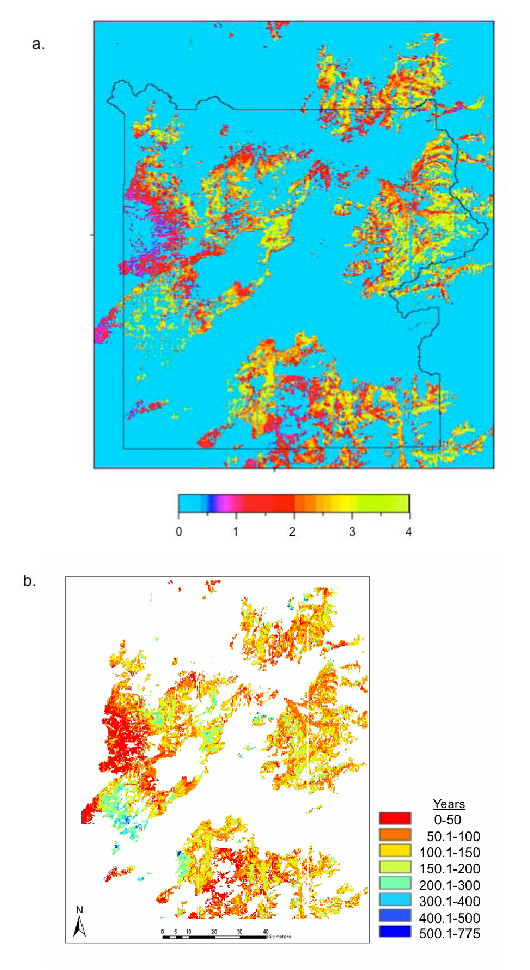
**(a) Ratio of CASA's default prediction for down CWD to the actual pools of down CWD reported by Huang et al. (2009b) in 2003-2007, and (b) Ratio of actual pools of total CWD reported by Huang et al. (2009b) to CASA annual NPP fluxes**.

The gridded map products of Huang et al. [46] for carbon in down CWD fractions in contact with soil surface were used to reset the "default" decomposing wood pool in CASA model runs starting in the year 2000. The default algorithm in CASA estimated CWD amounts with inputs proportional to annual NPP of live wood biomass, this assuming in the absence of residual biomass pools left in place by loss of the previous forest stands to fires. The ratio of CASA's default prediction for down CWD to the actual pools of down CWD reported by Huang et al. [46] was computed (Figure 5a). This result indicated that 2-4 times more biomass in decomposing wood had actually fallen to the ground in burned forest areas of the central and eastern portions of YNP (particularly in the Clover-Mist and Storm Creek Fire areas) since 1988 than was predicted by CASA default algorithms for CWD pools in these same areas. Our comparison of CASA default predictions to the radar-hyperspectral fusion product for down CWD also suggested that burned forest areas from 1988 on the western boundary of YNP (within the North Fork Fire area) have very little residual CWD remaining from the former forest stands that were growing in those areas.

#### Predicted net ecosystem carbon fluxes

Examined by landscape areas, mean annual NEP was highest (net sink greater than +50 g C m^-2 ^yr^-1^) in Hayden Valley, on southern shores of Yellowstone Lake, and in the Falls River Basin in the extreme southwest corner of YNP (Figure 4b), all of which were not burned during the 1988 fires. Mean annual NEP was lowest (in excess of -100 g C m^-2 ^yr^-1^) on the Madison and Pitchstone Plateaus and in the northeast sections of the North Fork Fire area. These areas of lowest annual NEP were characterized by low annual NPP (less than 120 g C m^-2 ^yr^-1 ^; Figure 4a) and relatively high amounts of CWD still in contact with the soil surface (Figure 5a)

Summarized by vegetation classes, CASA predicted NEP per unit area (m^2^) was a net source flux of CO_2 _to the atmosphere only for shrubland (sagebrush) areas in YNP, with a mean value of -22 g C m^-2 ^yr^-1 ^(Table 2a). All other vegetation classes were estimated as net ecosystem sinks of atmospheric CO_2 _on annual basis, with the predicted NEP for evergreen forest at a mean value of +47 g C m^-2 ^yr^-1^, making the entire study area a moderate net sink for atmospheric CO_2 _about +0.13 Tg C yr^-1^.

**Table 2:** CASA annual net ecosystem production (NEP) for the Yellowstone study area by (a) vegetation classes and (b) 1988 burned areas.

Class Name	Area (m^2^)	Minimum	Maximum	Mean	Standard Deviation
a. NLCD Vegetation					
Barren	5.33E+07	-188.5	191.4	22.6	36.7
Evergreen Forest	6.25E+09	-162.3	148.5	46.7	45.3
Shrubland	5.90E+09	-195.8	158.4	-21.9	40.7
Mixed Forest	6.25E+04	-77.0	92.5	5.3	20.7
Cultivation	1.33E+07	-158.6	112.8	-3.5	40.5
Wooded Grassland	9.03E+07	40.6	77.8	56.9	15.5
Deciduous Forest	1.88E+05	-163.2	217.6	4.9	41.8
TOTAL Land	1.42E+10			14.0	
					
b. FIRE Area 1988					
North Fork	2.14E+09	-162.1	164.1	-7.8	46.9
Clover-Mist	1.17E+09	-162.8	140.3	-12.4	40.0
Storm Creek	5.09E+08	-124.1	149.4	-1.0	37.5
Snake	4.68E+08	-144.9	179.3	-10.1	46.1
Mink	4.23E+08	-161.3	158.4	-2.7	43.9
Hellroaring	4.13E+08	-147.2	160.2	3.7	41.8
Huck	2.33E+08	-135.7	165.2	-6.8	43.2
Red-Shoshone	1.39E+08	-124.2	167.1	8.9	46.2
Clover	1.28E+08	-142.6	93.5	-13.6	36.4
Shoshone	1.28E+08	-162.3	128.5	-9.8	36.0
Fan	1.11E+08	-151.7	117.7	-5.6	47.1
Red	9.89E+07	-137.3	114.7	-11.5	48.5
Falls	6.60E+07	-134.2	108.4	-21.1	41.9
Mist	1.94E+07	-163.2	95.6	-16.7	40.6
Emerald	5.38E+06	-86.0	70.0	-16.9	40.8
Stormcreek	2.31E+06	-79.6	65.4	-17.4	35.0
TOTAL Burned Land	6.05E+09			-7.1	

All NEP estimates are in units of g C m^2 ^yr^-1^, with negative values representing net source fluxes of CO_2 _to the atmosphere and positive values representing net sink fluxes of CO_2 _from the atmosphere. Fires are sorted by total area burned.

This positive average NEP sink value for forested lands can nevertheless mask the contribution of forests burned during the 1988 wildfires, which were estimated as net source areas for CO_2 _with an overall mean value of -7 g C m^-2 ^yr^-1 ^and a total NEP flux of -0.04 Tg C yr^-1 ^for the entire burned area. The burned areas with the largest net sources of CO_2 _were (in order of mean NEP values) the Falls Fire, the Storm Creek Fire, and the Clover-Mist Fires, with a range of -21 to -12 g C m^-2 ^yr^-1 ^as net source fluxes of CO_2 _from the decomposition of CWD remaining in contact with the ground from the 1988 wildfires (Table 2b). We could also identify many sub-areas (all greater than 350 hectares) within these burns with NEP fluxes in excess of -75 g C m^-2 ^yr^-1^, and isolated areas with NEP fluxes in excess of -150 g C m^-2 ^yr^-1^.

**Figure 6:**
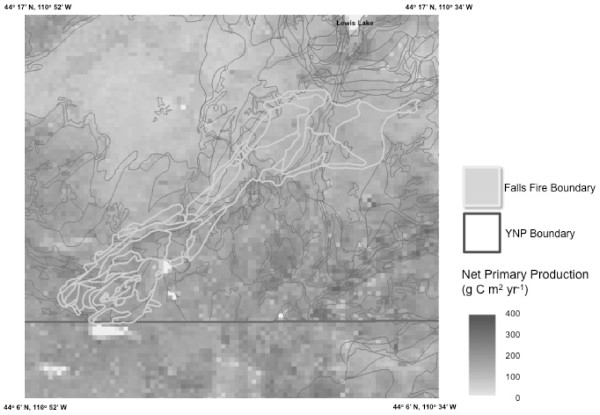
**CASA predicted NPP in the Falls Fire area of Yellowstone National Park**.

To further examine the impacts of the 1988 wildfires on forest carbon cycling, we focused more closely on the Falls Fire area, which had the lowest mean NPP and the highest NEP source fluxes from the CASA model of any major fire area in YNP (Table 2b). This burn was located in the southern section of YNP between 4 and 8 km south of Lewis Lake (Figure 6). The Falls Fire extended in a southwestern direction from 44°14' N, 110°35' W to 44°8' N, 110°50' W with a width of 3-4 km. The burned area was dominated by middle- and late- successional lodgepole pine stands prior to the 1988 fires [5]. Total aboveground stem biomass for these mature forest stands prior to 1988 would have represented around 80 tons (10^6 ^g) C ha^-1 ^[16]. Consistent with these pre-fire conditions, the post-fire pools of CWD mapped from airborne remote sensing by Huang et al. [46] for the Falls Fire were estimated at between 50-100 tons C ha^-1 ^in the northern portions of the burned area and less than 50 tons C ha^-1 ^in the southern portions.

CASA model predictions for annual NEP were typically source fluxes of between -50 and -75 g C m^-2 ^yr^-1 ^in the northern portions of the burned area and between 0 and -50 g C m^-2 ^yr^-1 ^in the southern portions of the Fall Fire area. These NEP flux patterns followed a drop in elevation from 2550 to 2200 meters, along with a gradient of average annual NPP of 120 g C m^-2 ^yr^-1 ^in the northern portions of the burned area to NPP of 200 g C m^-2 ^yr^-1 ^in the southern portions. CASA modeling identified large northern sections (around 44°14' N, 110°37' W) of the Falls Fire burn that were late-successional lodgepole pine stands prior to 1988, which appeared to be regrowing slowly from the fire effects, based on NPP model predictions circa 2006. At current NPP rates of growth (Figure 6), we can project linearly that it would take nearly 135 years to reestablish the aboveground carbon pools of middle- and late- successional lodgepole pine stands in much of the Falls Fire area.

## Discussion

A wide range in elevational gradients, precipitation patterns, soil types, fire histories, and wildlife management policies have made YNP a complex landscape in which to understand ecosystem carbon cycles. A protected area as large and inaccessible as YNP presents many challenges to ground-based measurement efforts. Fortunately, remote sensing using a combination of airborne and satellite platforms, has started to capture many of these co-varying spatial factors over the study area. Combined with process simulation models like CASA, many important observational constraints can be brought to bear on ecosystem production estimates.

A prime example (shown in Figure 5b) is the combination of actual pool sizes of post-1988 CWD detected by Huang et al. [46] from fusion of airborne imagery to our CASA mean annual NPP fluxes (years 2000-2006) computed from MODIS satellite images. The result of this combination ratio is a map, in units of years, that represents the time necessary to regrow every forest stand in YNP to its mature (pre-1988 fire) standing wood biomass. Since the current pool sizes of total CWD from remote sensing would be just slightly lower than the pre-1988 standing biomass amounts (due to combustion losses; [17]), and CASA annual NPP in units of carbon flux is roughly proportional (50% fraction; [30]) to the amount added to aboveground stem wood pools each year, a comparison of the two images generates a detailed forest regrowth product.

The shortest projected regrowth time was estimated at less than 50 years over the western portions of the North Fork Fire and parts of the Red-Shoshone Fire (Figure 5b); these areas were dominated by early-successional lodgepole pine stands prior to 1988 [5]. The burned areas with the longest projected regrowth time, estimated at more 200 years, were located on the southern extreme of the North Fork Fire (Madison Plateau) and in the Shoshone Fire areas, where middle- and late-successional lodgepole pine stands dominated prior to 1988 [5]. Most of the other burned areas in YNP were assigned projected regrowth times of between 50 and 200 years.

Crabtree et al. [13] reported that CASA predicted NPP increases with successional stage of (lodgepole) pine the most common cover type in YNP, but drops back down in the mature/climax stage to the young, post-disturbance levels of NPP. As stand age increases, mature lodgepole pine cover types may experience relatively higher autotrophic respiration rates during the growing season, compared to younger age stands characterized by smaller bole diameters and lower metabolic baselines [50]. The successional pattern of CASA NPP in lodgepole pine stands, as well as the negative correlation between fire severity index and NPP in the spatial autocorrelation model of Crabtree et al. [13], supports the hypothesis that an area subjected to a burn will have reduced NPP in the short-term, then will slowly recover to pre-burn NPP levels, followed by a gradual decrease in NPP during later successional stages.

An overarching question is whether there will be long-term loss of carbon from forest areas burned in the 1988 Yellowstone fires. Ryan et al. [47] reported on chronosequence measurements of carbon pools (live and dead wood) in 77 stands in western YNP, replicated across age and tree density. The conclusions of this work were that carbon in subalpine forests of YNP recovers 'quickly' (within 50 years) after a fire, regardless of tree density. It was hypothesized that, if a forest in YNP replaces itself after disturbance, there will be no long-term loss of carbon from the ecosystem. Kashian et al. [15] submitted that wildfire is unlikely to change carbon stored in forests by more than 10%, unless fire converts forests to grasslands.

The uncontrolled variable in these inventory-based calculations, however, is climate change. McMenamin et al. [51] reported trends in Yellowstone weather station records since 1949 that showed increases in average spring (March-May) and summer (June-August) temperatures. Over the same period, stations reported an increase in maximum annual temperature, and a decrease in total annual precipitation. The decrease in precipitation was most severe during the winter months (December, January, and February) and has lead to a reduction in snowpack, as well as notable wetland desiccation. In the years since 2000, YNP has experienced the most severe droughts on record, dating back over 100 years.

Climate change impacts on C and N fluxes among mature and regenerating lodgepole pine stands in YNP were modeled by Smithwick et al. [52]. Their results could not be seen as definitive, however, with total C storage predicted to be 0-9% higher under one climate model outcome, and 5-6% lower under another climate model outcome. Overall, this modeling experiment suggested that fire return intervals would need to be dramatically reduced (from around 300 years to 175 years) to affect long-term carbon storage in the Yellowstone ecosystem, due to large soil N pools and relatively fast recovery of aboveground C pools following fire.

## Conclusions

Ecosystem production and carbon fluxes in the Yellowstone region over the next century will likely reflect complex relationships between climate, forest age structure, and disturbance-recovery patterns of the landscape, plus management policies for large grazing herbivores and their predators. Ground-based measurement data sets for YNP have provided process-level understanding of carbon cycles that can help address this complex matrix of factors, aided by remote sensing of all areas of the Park. The need for further analysis of airborne and satellite observations at high spatial resolutions (< 100 m) of vegetation structure and recovery patterns is therefore a high priority for future research.

## Competing interests

The authors declare that they have no competing interests.

## Authors' contributions

All authors have made substantial contributions to the analysis and interpretation of data, have been involved in drafting the manuscript and revising it critically for important intellectual content, and have given final approval of the version to be published.
